# Effect of persuasive messages on National Health Service Organ Donor Registrations: a pragmatic quasi-randomised controlled trial with one million UK road taxpayers

**DOI:** 10.1186/s13063-018-2855-5

**Published:** 2018-09-21

**Authors:** Anna Sallis, Hugo Harper, Michael Sanders

**Affiliations:** 1 Strategy Group, Department of Health and Social Care, Richmond House, 79 Whitehall, London, SW1A 2NS UK; 2 Public Health England Behavioural Insights Team, Public Health England, Wellington House, 133-155 Waterloo Road, London, SE1 8UG UK; 3 The Behavioural Insights Team, 4 Matthew Parker Street, London, SW1H 9NP UK

**Keywords:** Organ donation, Social norms, Reciprocity, Message framing, Loss frame, Gain frame, Cognitive dissonance

## Abstract

**Background:**

A shortage of organs available for transplantation is causing loss of life. Increasing the number of individuals on the National Health Service (NHS) Organ Donor Register (ODR) is one way to address the shortage of organs. In Great Britain, new drivers registering for their driving licence are invited to join the ODR. A further 17 million drivers renew their road tax online each year, presenting an additional opportunity to prompt drivers to join the ODR. This trial explores the effect of adding persuasive messages to a prompt to join the ODR at the end of road tax payment transactions.

**Methods:**

In this pragmatic, parallel group, quasi-randomised controlled trial, drivers renewing their road tax or registering for a driving licence were alternately allocated, using a JavaScript randomisation code embedded in the GOV.UK website, to view a control prompt inviting sign-ups to the ODR or the same prompt plus one of seven theoretically informed persuasive messages; (i) social norms alone, (ii) social norms plus the NHS ODR logo, (iii) social norms plus an image, (iv) loss frame, (v) gain frame, (vi) reciprocity and (vii) cognitive dissonance. The trial took place over a 4-week period in June 2013. The primary outcome measure was participants completing the online registration form (sign-ups).

**Results:**

Altogether, 1,085,322 website users were included in the study. Further, 1171 more sign-ups were completed under the most effective message (reciprocity) compared to the control prompt alone (reciprocity: *n* = 4256, control: *n* = 3085; odds ratio, OR 1.38, 95% confidence interval 1.32–1.45, *p* < 0.001). The loss-framed message was as effective. All messages increased sign-ups compared to the control prompt apart from the social norms message plus image (*n* = 2879; OR 0.94, 95% confidence interval 0.89–0.99, *p* < 0.05).

**Conclusions:**

Short persuasive messages alongside a prompt can persuade more ODR sign-ups for individuals renewing their road tax than a prompt alone. The most effective message remains in place today. Since the trial in 2013, the same message has been implemented across 25 government end-of-transaction websites on GOV.UK, resulting in 529,000 new registrations to the ODR up to 31st October 2017.

**Electronic supplementary material:**

The online version of this article (10.1186/s13063-018-2855-5) contains supplementary material, which is available to authorized users.

## Background

Organ transplantation can save the lives of patients with organ failure and is one of the most revolutionary advances in human medicine in the modern era. However, to save lives, organ transplantation relies on the availability and suitability of donated human organs from both living and deceased donors. As of March 2017, there were 6388 people on the transplant waiting list in the UK and 457 patients died in the previous 12 months while on the active waiting list [1]. One way to increase the number of organs available for transplant is to increase the number of people on the National Health Service (NHS) Organ Donor Register (ODR). The ODR is a national, confidential list of individuals living in the UK who are willing to become organ donors after their death. In March 2017, the total number of individuals on the ODR was 23.6 million (36% of the population) [1]. Evidence consistently suggests favourable opinions towards organ donation (e.g. [2, 3]) and yet only around a third of the UK population is on the ODR. Given this finding, an approach aimed at turning favourable attitudes into action by increasing the opportunities available to join the ODR seems sensible. Indeed Falomir-Pichastor et al. [3] noted in their review of factors influencing organ donation that attitudes are so favourable towards organ donation that simply asking people to sign a donor card can increase sign-up rates, leading them to question whether there is a need to persuade people to join or whether the focus should be on increasing opportunities to invite people to join the ODR.

In Great Britain, individuals applying for a driving licence are mandated to answer a question about whether they wish to donate their organs after death. The largest number of registrations (58% in 2016–17) has been achieved through this route via the Driving and Vehicle Licencing Agency (DVLA) website [1]. Such message immediacy approaches work by exposing individuals to messages that link decisions closely with the desired behaviour (joining the ODR) in an environment where the behaviour can be easily and immediately enacted. Similar point of decision materials, when used for an intervention at the Department of Motor Vehicle offices in Michigan, almost doubled registrations [4]. The authors concluded that message immediacy can be an effective method to increase the desired behaviour but, to enhance the approach, the use of theoretically informed message variants should be considered.

Other current opportunities to join the ODR involve prompted choice interventions, for example inviting individuals to join the ODR when registering for a Boots (large chain of pharmacies) Advantage card (rewards point shopping card) and when registering at a general practice. Additionally, in the UK, NHS Blood and Transplant (NHSBT) regularly produce mass media campaigns and appeals. After an intense period of televised mass media campaigns, it is possible to observe and logically attribute large numbers of additional registrations to the success of these campaigns. A meta-analytic review of organ donation communications appeals found that, across 23 campaigns, there was an average 5% increase in study outcomes (i.e. registry signing) compared to control groups [5]. The effective components of these campaigns are difficult to isolate (and therefore replicate) in later campaigns, appeals and interventions. Experimental studies are required for this. In one such study, Siegel et al. [6] tested four different persuasive messages to increase organ donor registrations in the US general public across four settings. The counter-argument appeal, refuting common organ donation myths, proved more effective than appeals based on emotions, motivating action and highlighting dissonance, apart from in a hospital setting where emotional appeals were more successful. However, it should be noted that a range of other behaviour change techniques could also be identified in the materials that did not always pertain to the described intervention arm or were not consistently present across the format variations. For example, all intervention materials additionally contained a loss-framed message, ‘17 people die each day’, which may have had differential interactive effects on the message variants. Also, a call to action was present in some conditions and not others. It is, therefore, not possible to determine reliably from this trial which individual theoretical component of the messages worked best at increasing registrations.

Given the current reliance on prompted choice interventions and media campaigns to increase ODR sign-ups and the lack of evidence for message content, it is important that robust evidence is generated on the impact of such activities and to inform the design of future interventions. An opportunity to prompt individuals to join the ODR after applying for vehicle road tax (in addition to the existing DVLA prompt for individuals applying for a driving licence) arose through the Government Digital Service (GDS) and the GOV.UK website. The large number of individuals using GOV.UK to renew their road tax or register for a driving licence online (17 million per year) means that even a small increase in the number of registrations could result in large numbers of additional ODR registrations. A collaboration was formed between the Behavioural Insights Team, the UK Department of Health, NHSBT, GDS and the DVLA to test whether and which theoretically informed persuasive messages added to a prompt to join the ODR improves ODR sign-ups.

## Methods

### Study design

The trial employed a pragmatic, parallel group, quasi-randomised (equal ratio alternate allocation) controlled design with a control arm and seven intervention arms: (i) social norms alone, (ii) social norms plus logo, (iii) social norms plus image, (iv) loss frame, (v) gain frame, (vi) reciprocity and (vii) cognitive dissonance.

### Participants, randomisation and data collection

All members of the public in England, Scotland and Wales who renewed their vehicle tax or registered for a driving licence online between 24 June 2013 and 19 July 2013 were quasi-randomly assigned to see one of eight variants of a web page on GOV.UK at the end of their transactions. Each web user was asked to join the ODR by clicking a ‘join’ button, which directed them to the ODR registration page where they could complete a short online form to register. Alternatively, participants could click to ‘Find out more’, which took them to the NHSBT home page. Participants were also free to close the web page as their previous transaction was complete.

A JavaScript randomisation code embedded in the GOV.UK web page sequentially assigned individuals a number from 0 to 7 and displayed one of eight corresponding web pages. The first participant was assigned to control, the second to social norms alone and so on. Given that participants were blind to being in a trial, and thousands visited the page each day, this assignment approximates randomness insofar as there is no reason to suspect that assignment is correlated with any participant characteristics. GDS collected data on how many participants were assigned to each web page variant, and the day and hour they visited. NHSBT collected data on the website variant of origin (and hence intervention number) of individuals arriving at their registration page, and whether those individuals went on to register.

Note that those who were registering for a driving licence on the DVLA website were mandated to answer a question about joining the ODR as an integrated part of the process before reaching the final GOV.UK page, where this intervention message asks them again. This earlier request is not part of the trial and makes up around 10% of observations. Due to data protection protocols, we were unable to separate this group from those renewing their road tax on GOV.UK, who were invited only once. No data on age, sex or residing country were available for stratification or analysis.

The research was reviewed at regular project steering groups and undertaken with agreement from all stakeholder organisations, including the UK Cabinet Office, GDS, DVLA, the Department of Health and Social Care, and NHSBT. Alterations to web pages and messaging are routinely tested by GDS and this was considered part of the normal operating procedure. NHSBT are responsible for all national communications on organ donation. NHSBT reviewed, amended and approved all messages and web page content. No personally identifiable data were shared with project staff.

### Interventions

Seven theoretically informed persuasive messages alongside a prompt to join the ODR were tested against a prompt only control (with no persuasive message). All web page variants had the same formatting as the control but included an additional persuasive message (see Table 1 for message content and Additional file 1 for a screenshot of each message). As far as possible, the sentence length was consistent across messages and a single theoretical concept was used within each message to avoid interaction effects. Authors were restricted in terms of strictly testing theoretical message content because the priority was to test messages deemed acceptable to the public, sensitive to individuals on the transplant waiting list and which were not misleading. As such, some of the message content had been used in previous NHSBT campaigns. Messages were kept short, as it was assumed participants would have a reduced attention span following completion of an online form.

**Table 1:** Intervention messages

Intervention arm	Persuasive message
Control	No message
Social norms	Every day thousands of people who see this page decide to register.
Social norms plus logo	Every day thousands of people who see this page decide to register. (Plus logo)
Social norms plus image	Every day thousands of people who see this page decide to register. (Plus image)
Loss frame	Three people die every day because there are not enough organs.
Gain frame	You could save or transform up to 9 lives as an organ donor.
Reciprocity	If you needed an organ transplant would you have one? If so please help others.
Cognitive dissonance	If you support organ donation please turn your support into action.

#### Social norms

Social norms are rules or standards that are understood by members of a group or a society and guide or constrain social behaviour [7]. Knowing what others do guides our perception of what we think is normal and therefore what we should do [7]. Normative beliefs have been found to be important for increasing positive attitudes towards organ donation [3] and social norms have proven to be persuasive in changing behaviour in many areas, such as energy efficiency [8, 9], littering [1], charitable giving [11] and tax compliance [12]. Given the success of social norms applied to behaviour change in other areas and the lack of experimental evidence for this concept in organ donation, the first message targeted descriptive norms.

The message provides information about what others in the same context (those who viewed the web page during a pilot construction) had done: ‘Every day thousands of people who see this page decide to register.’ The message was designed to avoid stating a minority social norm (e.g. only a third of people are registered on the ODR), as this has been shown to reverse the effect [13]. This message was presented alone, with the NHS ODR logo or with an image. These variants were aimed at increasing message salience by using visual cues alongside the written text. It was speculated that an official logo (a picture of a heart) would increase the legitimacy of the message content, which is particularly relevant in a digital setting. This variant also has immediate practical application for the content of NHSBT marketing practices in similar contexts. In the final social norms variant, a photograph alongside the message shows a group of smiling individuals, ranging in age, sex and ethnicity. The group is intended to represent the diversity of people who have joined the register and encourage people to think that the social norms message applies to them (i.e. that people like them, as shown in the photograph, have also joined the register). In previous work, photographs increased the effectiveness of testimonials encouraging charitable giving [10, 14].

#### Loss- and gain-framed messaging

The origins of positively and negatively framed messaging are in prospect theory [15, 16], where it is proposed that we avoid risk for gains but we will take risks to avoid loss. The theory suggests that losses are felt more strongly than equivalent gains and the outcome is less important than the perceived value of the loss or gain [16]. Whether a behaviour is believed to be considered risky, uncertain and or probable is important. In health, there is an accumulation of evidence that for detection behaviours, such as breast screening, loss-framed messages facilitate action as they are considered risky. For illness prevention or protective behaviours, such as using sun screen, gain-framed messages are best at prompting action, as they are considered less risky [17]. A more recent meta-analytic review similarly found evidence for gain frames working for protection and prevention behaviours but there was less evidence for the effect of loss frames working for detection behaviours, unless a perception of high risk was attached to the health behaviour in question [18].

This body of research evidence is based upon losses or gains for the individual, unlike in organ donation where the loss or gain will be felt by another person. In the US, Reinhart et al. [19] found that students exposed to gain-framed organ donation campaigns were more likely to respond positively compared to those exposed to loss-framed messages. They also measured psychological reactance, which occurs when an individual perceives their beliefs and behaviours are threatened and their freedom to enact them is restricted [20], and perceived manipulative intent, which refers to the belief that the messenger is attempting to manipulate the reader into an action, such as buying a product or in this case signing the ODR. Responses were mediated by both psychological reactance and perceived manipulative intent and both were greater for the loss-framed message. However, where an individual had a prior intention to become an organ donor, individuals exposed to the loss-framed message experienced lower psychological reactance and perceived manipulative intent suggesting that a loss-framed message could be effective if individuals hold positive attitudes to joining the ODR, as is the case in the UK. This finding requires further investigation. Therefore, the next two variants tested loss- and gain-framed messages.

The loss-framed message sensitively informed people of an undesirable outcome that could be avoided in terms of lives lost, a message which has been used by NHSBT in previous campaigns: ‘Three people die every day because there are not enough organs.’ The gain-framed message informed people of a desirable outcome that can be attained in terms of lives saved, again derived from aspects of existing NHSBT messaging: ‘You could save or transform up to 9 lives as an organ donor.’ The gain-framed message is more personalised, using the word ‘you’, while the loss-framed message avoids this direct personalisation to limit any psychological reactance and perceived manipulative intent.

#### Reciprocity

Reciprocity is an important concept for organ donation and wider donation work [21] and has previously been used to increase charitable donations [14, 22]. Internationally, in organ donation, different types of reciprocity have been both debated and implemented, such as financial incentives, tax breaks and preferential allocation for those committed to donating their own organs [23–25]. Israel, for example, operates a system whereby priority goes to those who have been on its register for more than 3 years and whose family members have become a donor. In the UK, people are currently listed for transplant and organs are allocated according to clinical need with a balance of ‘equity, utility, benefit and fairness’ [26]. The concept of anonymous voluntary reciprocal altruism was applied to organ donation by Landry. In a feasibility test, medical students were asked if they would have an organ transplant to save their lives and were then asked whether they would be willing to donate their organs after death. The baseline agreement to donate organs was 59% but this rose to 94% once a strong reciprocity proposition was included that allowed individuals to choose to donate only to others on the register [21]. This provides further support for using reciprocity to increase organ donation registrations in other contexts. Therefore, the next message tested is based upon reciprocity: ‘If you needed an organ transplant would you have one? If so please help others,’ which makes salient the relationship between the addressee as a potential donor and the addressee as a potential organ recipient, suggesting a reciprocal relationship between the two roles.

#### Cognitive dissonance

As previously discussed, we know there is a discrepancy between support or favourable attitudes towards organ donation and actual organ donor registration. Highlighting differences between individuals’ beliefs and actions has been shown to change behaviours related to exercise, sexual health and smoking [27, 28]. Individuals are motivated into action to resolve the psychological discomfort arising from their conflicting beliefs and behaviour. The final message aimed to tap into this desire for self-consistency by suggesting: ‘If you support organ donation please turn your support into action.’

### Outcome measures

The primary outcome measure was completed registrations (sign-ups). The secondary exploratory outcome measure was clicks to join the ODR, regardless of whether they went on to complete the registration process. The term ‘sign-up’ is used instead of ‘registrations’ as NHSBT consider registrations to mean new registrations (i.e. excluding duplicates, as some people may sign up more than once). This study did not explore whether sign-ups were in fact new registrations or duplicates.

### Power calculation

A power calculation was conducted in R before the launch of the trial. Initial discussions with trial partners suggested that approximately 800,000 observations would be obtained during the 4-week trial period (100,000 per condition). Based on a two-sided test of proportions, an effect of Cohen’s *h* = 0.016 with 95% statistical power could be detected. Based on an historic registration rate of 2%, this is equivalent to an absolute increase (or decrease) of 0.2 percentage points, or a 10% relative rise in the outcome measure.

### Data preparation and statistical analysis

Data were received from GDS and NHSBT separately. Data were merged in Stata according to the day, hour and condition and expanded to create individual-level data in which each observation relates to a single unidentified user of the website.

All variables were categorical and the outcome measures binary. The primary and secondary outcome variables were whether participants completed ODR registration (sign-ups yes or no) and whether they clicked through to the organ donation registration page (clicks to join yes or no). Other variables were the message variant they viewed (treatment condition 0–7), day of the week (1–7) and hour of arrival (1–24) on the web page. These were coded as ordinal categorical variables when received and decoded to a set of binaries (0 or 1).

Multiple logistic regression models were used to investigate associations between message variants and the outcome measures. The primary analysis explored sign-ups using two models: one in which the number of sign-ups is regressed on the full set of message variants, with the control condition as the reference category, and the second in which the day and hour the participant visited the site are included as covariates to rule out any effects caused by these factors. Day and time of day data were not available for 30 website users, who were excluded from the adjusted analysis. As day and hour did not materially change the results, the unadjusted analysis is presented for the full sample.

To understand whether the message variants had a differential impact on those clicking to join and signing up and those clicking to join but not signing up, the secondary binary outcome variable (clicks to join) is regressed on the full set of binary message variants using multiple logistic regressions. In the first model, the probability of clicking through to begin the sign-up process depending on message variant viewed is estimated for the entire sample. In the second model, we estimate the effects of message variant on actual sign-ups, that is, only for those participants who clicked to join.

## Results

### Descriptive statistics

**Fig. 1:**
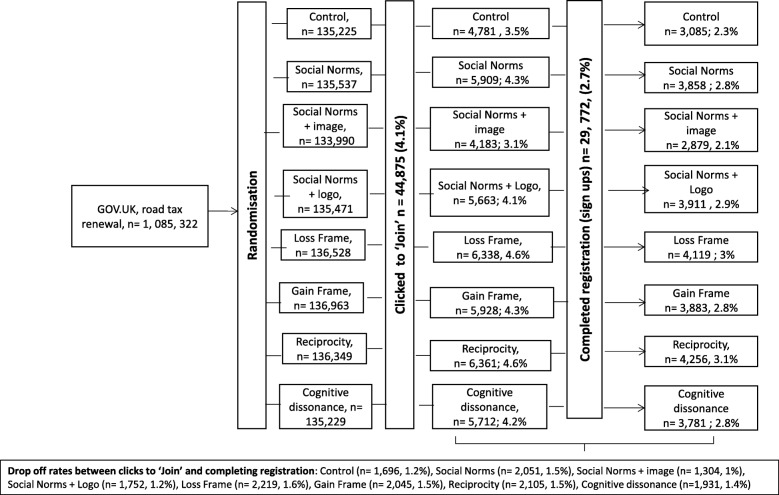
Participant flow through the trial

Data were recorded for all 1,085,322 participants who renewed their road tax or registered for a driving licence during the trial period. Figure 1 shows the number of participants assigned to see each message, the number of participants who clicked through to begin registration (clicks to join) and the number of participants completing registration (sign-ups), by message variant. Drop-outs between clicks to join and sign-ups are also displayed.

### Effects of persuasive messages on sign-ups to the ODR

The largest number of registrations were observed in the reciprocity condition, where individuals were 1.38 times more likely to register than if they had seen the control message (reciprocity odds ratio, OR 1.38, confidence interval, CI 1.32–1.45; *p* < 0.001). See Table 2. Registration is also more likely than the control with loss framing (loss frame OR 1.33, CI 1.27–1.40, *p* < 0.001), gain framing (gain frame OR 1.25, CI 1.19–1.31, *p* < 0.001) and cognitive dissonance messages (cognitive dissonance OR 1.23, CI 1.17–1.29, *p* < 0.001).

**Table 2:** Multiple logistic regression for effect of message variant on the primary outcome measure: ODR sign-ups

Outcome variable: Sign-ups to the ODR (0 did not sign up and 1 signed up)		
Message variants (reference: control message)	Unadjusted odds ratio [95% confidence interval]	Absolute effect (Percentage of sign-ups) (control group – 2.3%)
Social norms	1.255^***^ [1.196,1.317]	2.8%
Social norms and image	0.941^*^ [0.894,0.990]	2.1%
Social norms and logo	1.274^***^ [1.214,1.336]	2.9%
Loss frame	1.333^***^ [1.271,1.397]	3.0%
Gain frame	1.250^***^ [1.192,1.311]	2.8%
Reciprocity	1.380^***^ [1.317,1.447]	3.1%
Cognitive dissonance	1.232^***^ [1.174,1.293]	2.8%
Observations	1,085,322	1,085,322

*ODR* Organ Donor Register

^*^ *p* < 0.05, ^***^ *p* < 0.001

Participants viewing the social norms message were 1.25 times more likely to register compared to controls (OR 1.25, CI 1.20–1.31, *p* < 0.001). The addition of the NHSBT logo produces an effect nearly identical to presenting the social norms message alone (social norms and logo OR 1.27; CI 1.21–1.33, *p* < 0.001). The addition of an image, however, reduced registrations compared to control (social norms and image OR 0.94, CI 0.89–0.99, *p* < 0.05).

### Effects of persuasive messages on starting (clicks to join) and completing ODR registration (sign-ups)

Drop-outs at the two stages of registration were explored, firstly before clicking to join the register, where participants take no action as a result of seeing the message, and secondly after having clicked to join but before completing the registration process. Individuals viewing the loss-framed messages (OR 1.33, CI 1.28–1.38, *p* < 0.001) and reciprocity messages (OR 1.34, CI 1.29–1.38, *p* < 0.001) were most likely to click through compared to controls. See Table 3. Those who saw the social norms and image message were less likely than controls to click to join (OR 0.88, CI 0.84–0.92, *p* < 0.001). However, conditional upon having clicked to join (a sub-sample of all those who saw the messages), those who saw the social norms message with logo were most likely to actually register, compared to participants viewing the control message (OR 1.23, CI 1.13–1.33, *p* < 0.001). The overall poor performance of the social norms message with the image seems to be driven by fewer participants clicking to begin joining in the first place, because they were significantly more likely than controls to register, conditional upon having clicked through (OR 1.21, CI 1.11–1.32, *p* < 0.001).

**Table 3:** Multiple logistic regressions for effect of message variant on the secondary outcome measures, clicks to join (model 1), and sign-ups in those who had already clicked to join (model 2)

	(Model 1)	(Model 2)
	Unadjusted odds ratio for clicks to join (0 did not click and 1 did click)	Unadjusted odds ratio for completed registrations (sign-ups) in those who clicked to join, *N* = 44,875 (0 not completed and 1 completed)
Reference group (control)		
Social norms alone	1.244^***^ [1.197,1.293]	1.035 [0.956,1.121]
Social norms and image	0.879^***^ [0.843,0.917]	1.208^***^ [1.106,1.319]
Social norms and logo	1.191^***^ [1.145,1.238]	1.228^***^ [1.132,1.332]
Loss frame	1.329^***^ [1.279,1.380]	1.019 [0.942,1.102]
Gain frame	1.235^***^ [1.188,1.284]	1.048 [0.968,1.135]
Reciprocity	1.335^***^ [1.285,1.388]	1.114^**^ [1.029,1.205]
Cognitive dissonance	1.204^***^ [1.157,1.252]	1.084^*^ [1.000,1.175]
Observations	1,085,322	44,875

^*^ *p* < 0.05, ^**^ *p* < 0.01, ^***^ *p* < 0.001

Of the two messages most likely to result in overall sign-ups, individuals who had clicked to join after seeing the reciprocity message were signficantly more likely than the control group to complete registration (OR 1.14, CI 1.03-1.21, *p < 0.01)  *whilst those who clicked to join after viewing the loss framed message were not (OR 1.02, CI 0.94-1.10).

## Discussion

The two most effective messages at encouraging ODR sign-ups, in this context, are to offer people the chance to reciprocate and to make salient the loss of life entailed by too few organs being available for transplant. Those who viewed the reciprocity message and clicked to join were also more likely to complete the registration. The reciprocity message was, therefore, favoured by NHSBT and implemented immediately after the trial on the GOV.UK website at the end of road tax transactions and it remains in place at the time of writing. The same message has now been implemented across 25 government end-of-transaction web pages on GOV.UK, resulting in 529,000 new registrations since 2013 when the trial took place up to 31st October 2017.

Since a preliminary report of this study was published [29], the reciprocity message has been tested by other researchers with similarly positive effects. In Scotland, a similar reciprocity message increased intentions to join the ODR compared to controls, in particular when delivered online as opposed to face to face [30], providing further support for the use of the reciprocity message for online government platforms. Both sets of findings support Landry’s [21] feasibility study, making salient the potential reciprocal nature of the decision to join the ODR.

It is worth considering other possible hypotheses for the observed effect given the theoretical mechanism for its performance has not been confirmed and because the message will at some point become reduced in novelty and need to be updated. The reciprocity message was the only message posed as a question and might therefore have provoked more reflective thought, overcoming any immediately felt negative affect such as bodily integrity, medical mistrust, and the ick and jinx factors [31, 32]. Alternatively, it could have generated the question-behaviour effect, where merely asking a question about intention can encourage favourable action towards the behaviour in question [33]. Other explanations might be that the reciprocity message worked through inducing anticipated guilt, which has been shown to be an important predictor of behaviour in bone marrow donation [20, 34].

Both the loss- and gain-framed messages were more effective than the control message. The loss-framed message was as effective as the reciprocity message at encouraging participants to register, but this was largely driven by initial clicks to join. The drop-off rate between clicks to join and actually completing registration was lower than for other conditions. In a study by Reinhart et al. [19], although gain-framed messages encouraged organ donation intention and behaviours more so than a loss-framed message, this was mediated by psychological reactance and prior positive intentions towards organ donation. Given the widespread positive attitudes of the UK population towards organ donation, we can hypothesise that lower psychological reactance and perceived manipulative intent occurred in response to the loss-framed message compared to a population with less positive intentions. However, this does not explain the drop-off between initial clicks to join and actual registrations. One explanation is that the initial clicks to join are accounted for by more automatic prior positive beliefs about organ donation, which are overcome given time for reflective thought to allow feelings of psychological reactance and perceived manipulative intent to develop.

Another key aspect of loss aversion is risk. Research on organ donation behaviour has shown that individuals weigh up the potential benefits and risks to themselves of posthumous organ donation (e.g. body mutilation and inadequate medical care) and not just the benefits and risks to the potential recipient [35]. Cohen investigated the impact of loss-framed messaging (death of potential donor recipient) versus gain framing (survival of donor recipient) on willingness to sign an organ donation card in America [36]. Prospect theory suggests that loss-framed messages would work best here, as the behaviour would be considered risk laden and we act to avoid loss; however, no main effects were observed. Conversely, Cohen found that individuals perceiving there was a low risk responded better to loss-framed messages. Cohen also observed no difference in responses to loss- or gain-framed messages for high-risk individuals. The present study did not measure risk perception, but it is interesting to note that the loss- and gain-framed messages were not directly equivalent for reasons of appropriateness and acceptability and to avoid psychological reactance. Instead the loss-framed message referred solely to other people (‘Three people die every day…’), thereby potentially reducing the salience of the risk to oneself compared to the gain-framed message, which indicated that the impact of one’s own actions potentially increasing the personal salience of risk (‘You could save or transform up to 9 lives…’). If it were true that our loss-framed message evoked lower perceptions of personal risk, then our findings support Cohen’s unexpected findings that despite a perception of low personal risk, loss-framed messages can work to encourage organ donation, although this could be mediated by the level of personalisation in a message.

Three versions of a social norms message were tested. Of particular interest is the negative effect of adding a photograph of a group of people. This was the only variant to perform significantly worse than the control group, a result that cannot be attributed to the content of the social norms message, as when tested in isolation this performed significantly better than the control group. This effect did not generalise to the NHSBT logo, so it cannot be assumed that images in general undermine the effect of a message by distracting attention. It is possible that the photograph may have caused the web page to look like general marketing and therefore, indicated the end of the transaction, encouraging participants to close the page. The image also lacked specificity, context and relevance to the words. It was not clear if these people were awaiting transplants, if they were donors or if they were on the register. Another explanation in line with the first is that the image undermined the messenger, appearing as general marketing and therefore caused perceived manipulative intent and psychological reactance. Both concepts have been shown to mediate the impact of message framing and persuasion for organ donation [34]. The image may have increased the visibility of the messenger as an untrusted or unknown source, unlike the logo.

The primary outcome measure was completion of registrations, which required individuals to first click ‘Join now’ and then to proceed to complete the registration page. The loss-framed and reciprocity messages were similarly likely to motivate individuals to click ‘Join now’. However, those who saw the reciprocity message were significantly more likely to complete the registration process. This suggests a longer lasting effect on motivation. Additionally, this suggests that both treatments are equally powerful at attracting people to register, but that the reciprocity ask is more effective at overcoming friction costs and increasing motivation overall. It is also noteworthy that the negative impact of the social norms and people image is driven entirely through lack of initial clicks. The social norms and image and social norms and logo messages were actually most effective at translating clicks into registrations. This analysis is merely suggestive, as it is possible participants influenced by different messages may have differed on other important characteristics, for example their level of intrinsic motivation, their level of computer or internet literacy, or the amount of time they had available to complete the form and the study was not powered to detect differences for this secondary outcome.

The study had some limitations. The study tested sign-ups as opposed to new registrations. By this we mean that sometimes individuals who completed the form to join the ODR may have already been on the ODR. The study used alternate random allocation rather than pure randomisation, although with such a large sample size this is not expected to be problematic. Of the participants, 10% had already viewed a message encouraging them to join the ODR whilst registering for their driving licence and this group could not be separated in the analysis. Relatedly, it was not possible to determine the effects of country of residence or demographic variables, so the amount of variance explained by these factors is unknown. As a pragmatic field trial, aimed at finding out what works in a specific context, although messages were inspired by the theories described, without manipulation checks it is not possible to know if the mechanisms through which the messages operate can be fully attributed to these theories. This study did not look at additive or interactive effects for combining messages, or slight alterations in the phrasing of messages therefore we cannot be certain if the words or the posited theory worked. The loss- and gain-framed messages were not identical in content, since they refer to different outcomes. These messages were predominantly chosen because they were already in use by NHSBT in other marketing communications and other potential variants would have been nonsensical, insensitive or inaccurate. Likewise, the image used in the social norms variant was a photograph selected from a limited supply of images with existing copyright already used by NHSBT for promotional purposes. The results cannot be directly linked to the number of organ donations in the UK.

## Conclusions

This trial is an example of the importance of rigorously testing communications content before implementation. Without this, NHSBT could have inadvertently selected message content less effective than nothing at all. It is important to test the effectiveness of combining concepts within these messages, as well as looking to see if certain types of message resonate more with particular target groups for whom organs are in short supply.

The results of this trial provide the only evidence to date about which messages are most effective at motivating individuals to join the NHS ODR after renewing their road tax. As this trial was a natural field experiment with a large sample size, the external validity is high. This study represents a pivotal step in demonstrating the importance of robust testing of interventions that have the potential to impact upon large populations and in implementing the findings at scale and pace.

It is important to note that getting people to join the NHS ODR is one of the many issues around increasing the availability of organs for transplantation. The wider awareness raising of organisations like NHSBT is no doubt integral to the success of these prompted choice opportunities. It is particularly important that registrants on the ODR discuss their wishes with their family so that, should the time come when they could be an organ donor, their families are not taken by surprise and are willing to honour their loved one’s wishes.

## Additional file

Additional file 1: Screenshots of intervention messages. (a) Control. (b) Social norms. (c) Social norms and image. (d) Social norms and logo. (e) Loss frame. (f) Gain frame. (g) Reciprocity. (h) Cognitive dissonance. (DOCX 89 kb)

## Abbreviations

CI: Confidence interval
DVLA: Drivers and Vehicle Licencing Agency
GDS: Government Digital Service
NHSBT: National Health Service Blood and Transplant
NHS: National Health Service
ODR: Organ Donor Register
OR: Odds ratio
