# Supplementary material for: Effect of persuasive messages on National Health Service Organ Donor Registrations: a pragmatic quasi-randomised controlled trial with one million UK road taxpayers
Source: Trials. 2018 Sep 21;19:513. doi: 10.1186/s13063-018-2855-5 (PMC6150960; doi:10.1186/s13063-018-2855-5)

**Additional file 1: Screen shots of intervention messages:**

**A Control; B Social norms; C Social norms and image; D Social norms and logo; E Loss frame; F Gain frame; G Reciprocity; H Cognitive dissonance**


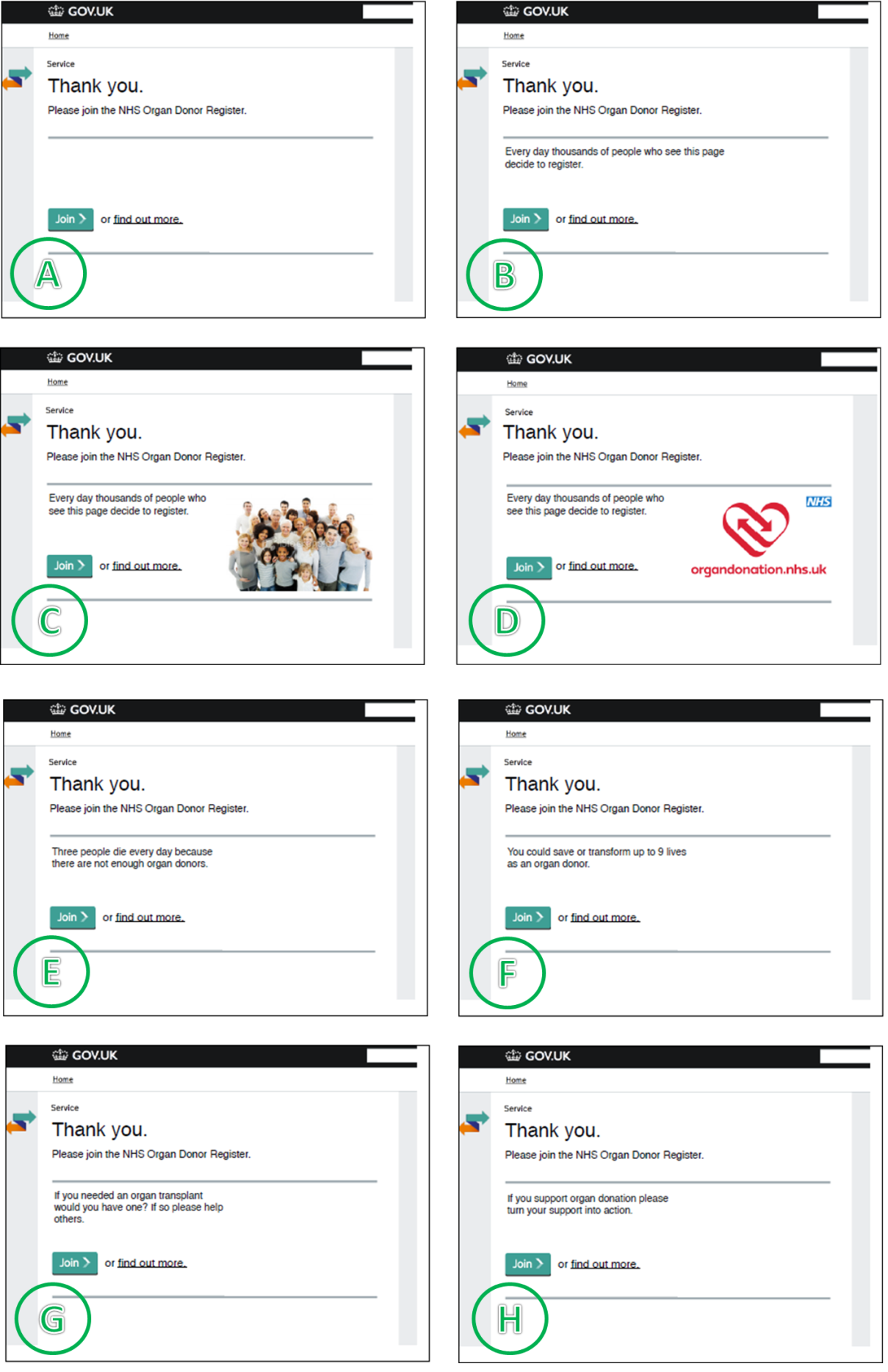

Supplement: Supplementary file 1 — Screenshots of intervention messages. (a) Control. (b) Social norms. (c) Social norms and image. (d) Social norms and logo. (e) Loss frame. (f) Gain frame. (g) Reciprocity. (h) Cognitive dissonance. (DOCX 89 kb) [file 13063_2018_2855_MOESM1_ESM.docx]
